# Mobile Phone Text Messaging Intervention for Cervical Cancer Screening: Changes in Knowledge and Behavior Pre-Post Intervention

**DOI:** 10.2196/jmir.3576

**Published:** 2014-08-27

**Authors:** Hee Yun Lee, Joseph S Koopmeiners, Taeho Greg Rhee, Victoria H Raveis, Jasjit S Ahluwalia

**Affiliations:** ^1^School of Social Work and University of Minnesota Masonic Cancer CenterCollege of Education and Human DevelopmentUniversity of Minnesota, Twin CitiesSt Paul, MNUnited States; ^2^Division of BiostatisticsSchool of Public HealthUniversity of Minnesota, Twin CitiesMinneapolis, MNUnited States; ^3^Division of Health Policy and ManagementSchool of Public HealthUniversity of Minnesota, Twin CitiesMinneapolis, MNUnited States; ^4^Psychosocial Research Unit on Health, Aging, and the CommunityCollege of DentistryNew York UniversityNew York, NYUnited States; ^5^Center for Health Equity and Department of MedicineSchool of MedicineUniversity of Minnesota, Twin CitiesMinneapolis, MNUnited States

**Keywords:** cervical cancer, Pap test, mobile health, text-messaging intervention, health behavior change, Korean American women, health disparity

## Abstract

**Background:**

Cervical cancer poses a significant threat to Korean American women, who are reported to have one of the highest cervical cancer mortality rates in the United States. Studies consistently report that Korean American women have the lowest Pap test screening rates across US ethnic groups.

**Objective:**

In response to the need to enhance cervical cancer screening in this vulnerable population, we developed and tested a 7-day mobile phone text message-based cervical cancer Screening (mScreening) intervention designed to promote the receipt of Pap tests by young Korean American women.

**Methods:**

We developed and assessed the acceptability and feasibility of a 1-week mScreening intervention to increase knowledge of cervical cancer screening, intent to receive screening, and the receipt of a Pap test. Fogg’s Behavior Model was the conceptual framework that guided the development of the mScreening intervention. A series of focus groups were conducted to inform the development of the intervention. The messages were individually tailored for each participant and delivered to them for a 7-day period at each participant’s preferred time. A quasi-experimental research design of 30 Korean American women aged 21 to 29 years was utilized with baseline, post (1 week after the completion of mScreening), and follow-up (3 months after the completion of mScreening) testing.

**Results:**

Findings revealed a significant increase in participants’ knowledge of cervical cancer (*P*<.001) and guidelines for cervical cancer screening (*P*=.006). A total of 23% (7/30) (95% CI 9.9-42.3) of the mScreening participants received a Pap test; 83% (25/30) of the participants expressed satisfaction with the intervention and 97% (29/30) reported that they would recommend the program to their friends, indicating excellent acceptability and feasibility of the intervention.

**Conclusions:**

This study provides evidence of the effectiveness and feasibility of the mScreening intervention. Mobile technology is a promising tool to increase both knowledge and receipt of cervical cancer screening. Given the widespread usage of mobile phones among young adults, a mobile phone-based health intervention could be a low-cost and effective method of reaching populations with low cervical cancer screening rates, using individually tailored messages that cover broad content areas and overcome restrictions to place and time of delivery.

## Introduction

Korean American women have one of the highest cervical cancer mortality rates in the United States. Cervical cancer incidence and mortality rates for Korean American women are roughly twice that of non-Latino white women [1]. While the *Healthy People 2020* initiative states that 93% of women, aged 21-65 years, should have undergone a Pap test within the past 3 years [2], studies consistently report that, among women across US racial/ethnic groups, Korean American women have the lowest Pap test screening rates, ranging from 39% to 64% [3-8]. Given that early detection of cervical malignancies through this routine screening measure has been shown to significantly reduce cervical cancer mortality, Korean American women’s low screening rate indicates that efforts to increase their screening behavior would be very beneficial [1,9].

A variety of structural and cultural factors act as barriers to screening for Korean American women. Structural obstacles include health access due to inadequate health insurance [10-12], expense [1,13], time constraints [10,13], and language limitations [1,10,12-15]. Cultural barriers to cervical cancer screening encompass lack of knowledge regarding cervical cancer and cervical cancer screening [1,10,12,13,16], a wrongly held belief that screening is unnecessary in the absence of symptoms or at young ages [1,10,12,13,16,17], cultural modesty or embarrassment [10,13,16], lack of culturally appropriate health care providers [12,13], and fear of receiving negative screening results [10,16].

A limited number of interventions to address barriers and promote cervical cancer screening among Korean American women have been designed or implemented. These efforts have focused on peer-led workshops [18,19], dissemination of videos [18], and distribution of cancer education print materials [20,21]. There are a number of reasons why these approaches have been only partially effective in promoting cervical cancer screening in this population. Korean American women are a particularly hard-to-reach population [19,20]. Although earlier interventions have specifically targeted structural barriers to cancer screening (eg, providing low-cost or free Pap tests or in-language services), prominent cultural obstacles such as cultural modesty or misconceptions about screening were not addressed [19]. These previous intervention strategies also did not tailor the intervention to strategically target specific individual concerns about screening, despite evidence that there are multiple cultural reasons for Korean American women’s reservations about cervical cancer screening [20]. The restricted scope and lack of tailoring in these previous interventions may have contributed to their limited impact. Personalized interventions may be necessary to motivate a change in screening behavior with this difficult to reach population.

To address the multiple limitations that were present in prior interventions, we developed and tested a mobile phone text message-based cervical cancer Screening (mScreening) intervention that utilizes mobile health (mHealth) technology. mHealth is defined as the use of mobile and wireless devices as intervention tools to deliver health information or improve health outcomes, particularly using short message service (SMS or text) and/or multimedia message service (MMS, or images or pictures) [22]. mHealth is considered a promising tool for preventive care through promotion of behavioral change. mHealth is taking a primary place in a number of research initiatives related to the promotion of health behavior. For example, mHealth technology was successfully used in weight management [23], smoking cessation [24-28], youth sexual health [29], increased physical activity [30], self-care behaviors [27,28,31], and asthma monitoring and management programs [32].

**Figure 1:**
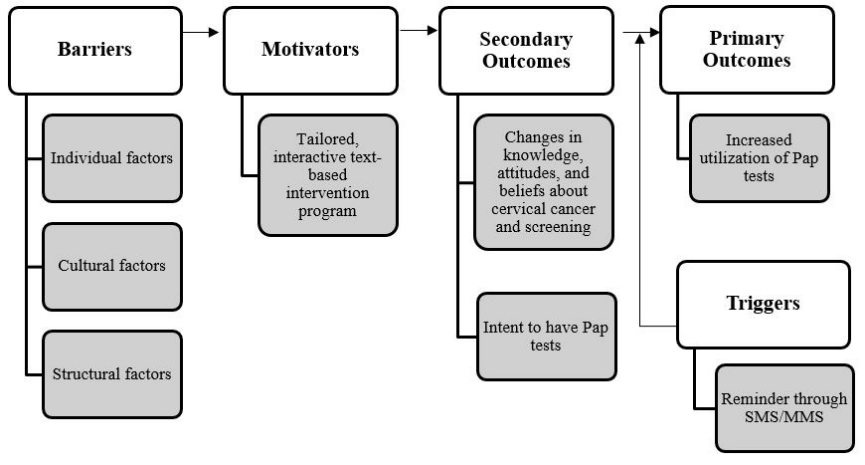
Conceptual framework of the study.

Our study seeks to harness mobile phone technology to positively influence cancer screening behavior, taking preventive health care approaches to a new level [7,33]. Guided by the Fogg’s Behavior Model (FBM) [33], the mScreening intervention consists of three sequential components: (1) identifying barriers, (2) developing motivators, and (3) providing triggers (see Figure 1 for conceptual framework). With the FBM framework, we first identified specific structural and cultural barriers that prevent Korean American women from receiving a Pap test. This information guided our subsequent development and implementation of mobile tools (eg, SMS or MMS) to improve knowledge and provide the motivation for behavioral change. Triggers to act are employed in the form of reminder text messages (eg, “Make an appointment now!”) or electronic links (eg, “Click to talk to a Korean health navigator”) to prompt Korean American women to take immediate action to obtain a Pap test.

Our study had three primary aims: (1) to examine if the mScreening intervention increased research participants’ (a) knowledge of cervical cancer, relevant guidelines of cervical cancer screening, and cervical cancer risk factors, and (b) their intent to undergo screening; (2) to assess if the mScreening intervention contributed to a 20% increase in the receipt of the Pap test over the sample’s baseline rate; and (3) to examine the acceptability and satisfaction of the 7-day mScreening intervention program. Our study used evidence-based and theory-driven approaches to develop the mScreening intervention to concentrate on barriers (eg, cultural beliefs, perceived-risk, and limited health literacy) that prior work had not addressed, so as to increase Korean American women’s adherence to cervical cancer screening guidelines.

## Methods

### Research Design and Data Collection

A quasi-experimental design was used with baseline, post-test (1 week after the completion of mScreening), and follow-up (3 months after the completion of mScreening) assessments. The eligibility criteria for our study included: Korean American women aged 21 to 29 years with no prior receipt of a Pap test in Minnesota. Participants also needed to have up-to-date health insurance, mobile phone access (either a regular or smartphone), and be familiar with or willing to learn basic elements of text-based information communication technology. We used a multi-pronged recruitment strategy. A variety of outreach flyers and brochures were posted and handed out in churches, clinics, social service agencies, and ethnic markets that serve the Korean American population, as well as advertised through public social media that is tailored towards the Korean American population. We fully explained the purpose of our study, eligibility criteria, confidentiality, and the voluntary nature of study participation to every participant both in oral and written formats before the study began. The University of Minnesota Institutional Review Board approved the research procedure for this study. A total of 30 participants were enrolled who met the inclusion criteria, received the intervention, and completed the data collection protocol. We had complete retention of participants in our study.

### Intervention

We used a community-based participatory research approach to develop the content of the 7-day text message-based mScreening intervention and participant recruitment strategies. We formed a community advisory board, which consisted of Korean American community leaders, health care professionals who work in the Korean American community, and directors of social service agencies. Additionally, we conducted a series of focus groups with 13 young Korean American women. After usability testing for the mScreening intervention with 8 Korean American women, we recruited 34 Korean American women and delivered the mScreening intervention to the 30 women who qualified as study participants.

As per the FBM [33], the mScreening intervention identifies individual barriers to screening, develops motivators specific to these barriers, and provides a trigger to the desired health behavior action (ie, receipt of a Pap test) (see Figure 1). The informational/educational content of the mScreening intervention covered the following topics: (1) introductory information on the cervix and cervical cancer, including statistical facts of cervical cancer incidence and mortality, and screening rates of cervical cancer among Korean American women compared to other racial/ethnic groups in the United States, (2) introduction of the Pap test as a preventive mode for cervical cancer, (3) information on health care accessibility, (4) introduction of cultural barriers, (5) availability of local clinics and cost of Pap test, (6) testimony of a Korean American woman who had gone through the Pap test experience, and (7) testimony of a Korean American cervical cancer survivor who found cervical cancer at later stage and had no previous receipt of a Pap test. The mScreening intervention was delivered to each participant over 20-30 minutes each day for a 7-day period at each participant’s preferred time.

The mScreening intervention contained a high level of interactive features, such as quizzes and questions, and also allowed participants to engage in conversation, with approximately one-third of messages asking for responses. Messages were individually tailored for each participant. For example, based on the baseline interview of each participant, we identified strengths and weaknesses of individual participants. If a participant had weaknesses on culture-based health beliefs on cervical cancer screening at baseline, we provided ad hoc messages that were designed to reduce cultural barriers (eg, “We understand it is a bit embarrassing to get it done. But do it for you! Your happy cervix will appreciate it!”) in addition to regular messages sent each day. This tailoring was enabled by a database of over 50 ad hoc messages that were designed to reduce culture-based beliefs and attitudes on cervical cancer screening.

### Measures

#### Overview

We collected information from participants through face-to-face interviews using a structured questionnaire administered at three time points: at the study enrollment (baseline), 1 week after completing the mScreening intervention (post-test), and 3 months after completing the mScreening intervention (follow-up).

#### Outcome Measures

Our primary outcomes of interest included knowledge, attitudes, and beliefs about cervical cancer and cervical cancer screening, intent to undergo screening as measured with the trans-theoretical model (eg, stages of change consisting of precontemplation, contemplation, preparation for action, action, and maintenance) [34,35], and the receipt of the Pap test.

#### Baseline Measures

We collected information on sociodemographic characteristics (eg, age, marital status, education, employment status, income, health status, and religion), health-related information (eg, history of cancer in the family, health insurance, usual source of care, and number of doctor visits), level of acculturation (years in the United States and English language proficiency), and administered scales for health knowledge and beliefs about cervical cancer, cervical cancer screening, and intent to undergo a Pap test as part of cervical cancer screening. Champion’s revised health belief model scale was used for measuring health beliefs on cervical cancer and cervical cancer screening [36]. This scale was previously used in Korean American women in Chicago and was demonstrated to have appropriate reliability and validity [1]. We adapted and used Taylor and colleague’s 15-item scale for knowledge about cervical cancer and cervical cancer screening [37].

#### Post-Test and Follow-Up Measures

One week after the completion of the mScreening intervention (post-test interview), we asked participants about their experience with the intervention and general satisfaction to inform quality assurance and improvement. We also repeated the items from the baseline interview about knowledge and beliefs about cervical cancer and cervical cancer screening, and assessed their intent post-test to undergo screening and if they received a Pap test after the intervention. In the 3-month follow-up interview, we asked participants if they had received a Pap test in the prior 3 months, and for those who did not receive a Pap test, we asked their reasons for not undergoing the test.

### Data Analyses

Differences in constructs relating to knowledge, attitudes, and beliefs about cervical cancer and cervical cancer screening from baseline to 1 week post-test were summarized by means and standard deviations, and tested using the paired *t* test. Two approaches for comparing the intent to receive the Pap test pre- and post-mScreening were considered. First, we translated the change in a subject’s intent to receive the Pap test to a numerical scale with subjects receiving a “1” if their intent to receive the Pap test increased from pre- to post-mScreening, a “0” if their intent to receive the Pap test stayed the same, and a “−1” if their intent to receive the Pap test decreased. We then summarized subjects’ change in their intent to receive the Pap test by the mean and standard deviation and compared the mean change to zero using the one-sample *t* test to determine if, on average, subjects’ intent to receive the Pap test increased from pre- to post-mScreening. In addition, we dichotomized intent to receive the Pap test into “intent to receive within a year” and “no intent to receive within a year”. The percent of subjects providing each response before and after mScreening were compared using McNemar’s test for paired binary data. We estimated the rate of cervical cancer screening post-intervention and at the 3-month follow-up using the sample proportion and 95% confidence intervals were estimated using the exact method. Measures of acceptability and satisfaction were summarized using counts and sample proportions. Given the preliminary nature of the study and relatively small sample size, multivariate analyses were not conducted and only univariate and bivariate results are reported.

## Results

### Demographic Characteristics of the Sample

Table 1 presents the study participants’ baseline demographic information (N=30). Participants ranged in age from 21-29 years, and 27 out of 30 participants (90%) reported living in the United States for less than 9 years, with 37% (11/30) living in the United States for 3 years or less. Of the 30 participants, 28 participants (93%) reported that they had health insurance; 15 participants (50%) reported having a primary hospital; and 6 participants (20%) reported having a primary physician. Finally, 19 participants (63%) reported a family history of cancer.

**Table 1:** Baseline sociodemographic characteristics of the sample (N=30).

Variable	Categories	n (%)
**Age, years**		
	21-22	11 (37)
	23-25	13 (43)
	26-29	6 (20)
**Marital status**		
	Married	1 (3)
	Other	29 (97)
**Years in United States**		
	3 or less	11 (37)
	4-8	16 (53)
	9 or more	3 (10)
**Employment**		
	Yes	11 (37)
	No	19 (63)
**Education**		
	Undergraduates	19 (63)
	Graduated from college/university	9 (30)
	Graduated from graduate school	2 (7)
**Monthly income (US$)**		
	under $499	14 (48)
	$500-$1499	10 (34)
	$1500 or more	5 (17)
**Health status**		
	Poor or fair	19 (63)
	Good	6 (20)
	Very good or excellent	5 (17)
**Living arrangement**		
	Live alone	12 (40)
	Live with spouse only	1 (3)
	Live with others	17 (57)
	Other	0 (0)
**Currently living in**		
	Rented house or condominium	19 (63)
	Government-subsidized senior citizen apartment	4 (13)
	Unsubsidized apartment	3 (10)
	Rented room in other’s home	3 (10)
	Other	1 (3)
**Self-rated financial status**		
	Very bad	1 (3)
	Bad	3 (10)
	Fair	20 (67)
	Good	4 (13)
	Very good	2 (7)
**How often do you work out per week?**		
	Not at all	8 (27)
	Once	2 (7)
	Twice	11 (37)
	Three times	4 (13)
	Four times or more	5 (17)
**Smoking status**		
	Not at all	28 (93)
	Some days	1 (3)
	Every day	1 (3)
**Alcohol consumption**		
	Not at all	13 (43)
	Some days	17 (57)
	Every day	0 (0)
**Health insurance**		
	Yes	28 (93)
	No	2 (7)
**Primary hospital**		
	Yes	15 (50)
	No	15 (50)
**Primary physician**		
	Yes	6 (20)
	No	24 (80)
**Family cancer history**		
	Yes	19 (63)
	No	11 (37)

### Changes in Knowledge, Attitudes, and Beliefs

Table 2 presents changes in measures of knowledge, attitudes, and beliefs about the Pap test, comparing baseline to the post-test, completed 1 week after the mScreening intervention. Significant differences were observed for all constructs and these differences remained significant after a Bonferonni multiple comparison adjustment (*P*<.01). Significant improvements were observed for general knowledge about cervical cancer (*P*<.001), knowledge about the Pap test (*P*<.001), beliefs about and attitudes toward the Pap test (*P*=.006), and knowledge about risk factors of cervical cancer and its screening (*P*<.001). We also observed a significant reduction in socio-cultural barriers to cervical cancer screening (*P*=.001).

**Table 2:** Changes in knowledge, attitudes, and beliefs about cervical cancer and the Pap test (N=30).

Construct	Baseline pretest	1 week posttest	Mean difference	*P* value
	mean (SD)	mean (SD)		
General knowledge about cervical cancer	0.33 (0.36)	0.92 (0.17)	0.59	<.001
Knowledge about Pap test	2.85 (0.87)	3.56 (0.52)	0.71	<.001
Beliefs of and attitude toward Pap test	2.83 (0.42)	3.14 (0.52)	0.31	.006
Knowledge about risk factors of cervical cancer and its screening	2.11 (0.80)	2.87 (0.57)	0.76	<.001
Socio-cultural barriers to cervical cancer screening	2.33 (0.51)	1.98 (0.53)	−0.35	.001

### Intent and Receipt of Pap Test

Table 3 presents the study participants’ intent to receive the Pap test before and after the mScreening program. We observed an increase in participants’ intent to receive the Pap test (mean 0.23, 95% CI −0.04 to 0.51) and the percent of participants indicating an intent to receive the Pap test within 1 year (from 63% to 87%) but these differences were not statistically significant (*P*=.090 and *P*=.070, respectively). In addition, one study participant (3%, 1/30) reported receiving the Pap test within 1 week after completing the mScreening program and 6 additional participants (20%, 6/30) reported receiving the Pap test by the 3-month follow-up visit, which represents a 23% (7/30) improvement in the proportion of participants receiving the Pap test (95% CI 10% to 42%) compared to baseline.

**Table 3:** Intention to receive the Pap test before and after the mScreening intervention (N=30).

Baseline pretest	Intent to receive a Pap test at 1 week post-test, n (%)					
	No plans within 1 year	Within 1 year	Within 3 months	Within 1 month	Have received Pap test	Total
No plans within 1 year	2 (6.7)	4 (13.3)	2 (6.7)	2 (6.7)	1 (3.3)	11 (36.7)
Within 1 year	2 (6.7)	9 (30.0)	1 (3.3)	1 (3.3)	0 (0.0)	13 (43.3)
Within 3 months	0 (0.0)	2 (6.7)	0 (0.0)	1 (3.3)	0 (0.0)	3 (10.0)
Within 1 month	0 (0.0)	1 (3.3)	0 (0.0)	2 (6.7)	0 (0.0)	3 (10.0)
Have received Pap test	0 (0.0)	0 (0.0)	0 (0.0)	0 (0.0)	0 (0.0)	0 (0.0)
Total	4 (13.4)	16 (53.3)	3 (10.0)	6 (20.0)	1 (3.3)	30 (100)

### Acceptability and Feasibility of the mScreening Intervention

Table 4 presents participant responses to questions relating to the acceptability and satisfaction of the 7-day mScreening intervention. At the 1-week post-test visit, 25 of 30 participants (83%) reported being either satisfied or very satisfied with the mScreening program and 29 participants (97%) reported that they would recommend mScreening to their friends.

**Table 4:** Acceptability and satisfaction of the mScreening intervention at 1-week post-test interview (N=30).

Question	Answer	n (%)
**Please rate your satisfaction level with mScreening program**		
	Very satisfied	8 (27)
	Satisfied	17 (57)
	Neutral	5 (17)
	Dissatisfied	0 (0)
	Very dissatisfied	0 (0)
**Would you like to recommend mScreening program to your friend?**		
	Yes	29 (97)
	No	1 (3)

## Discussion

### Principal Findings

We observed a significant increase in the study sample’s general knowledge about cervical cancer and the Pap test, beliefs and attitudes toward the Pap test, and knowledge about risk factors of cervical cancer and its screening guidelines (*P*=.006), after completing the mScreening intervention. We also found a significant decrease in perceived socio-cultural barriers to cervical cancer screening (*P*=.005). By the 3-month follow-up, 7 out of 30 participants (23%) reported having received the Pap test. As only women that had not previously received the Pap test were recruited for this study, this finding indicates that participation in the mScreening intervention led to more than a 20% increase in the receipt of the Pap test in this sample. This is a notable achievement as we targeted a group that had not previously been motivated to engage in this recommended health promotion activity. These findings are in line with a previous study, where an SMS reminder system resulted in a significant increase in the practice of breast self-examination [38].

Positive results were also obtained regarding the acceptability and satisfaction of the 7-day mScreening intervention program. At the 1-week post-test visit, 83% (25/30) of participants reported that they were either satisfied or very satisfied with the mScreening program, and 97% (29/30) of participants stated that they would recommend the mScreening program to their friends. Overall, the majority of participants expressed a high degree of acceptability and satisfaction with the mScreening intervention program.

Our study shows that the FBM [33] was useful in explaining and improving cervical cancer screening behaviors. We identified structural and cultural factors (eg, language limitations and lack of knowledge regarding cervical cancer and cervical cancer screening) as well as individual factors (eg, family history of cancer) that are barriers to cervical cancer screening in Korean American women. The mScreening intervention addressed such factors with the intention of minimizing the barriers and motivating research participants to act upon the preventive health behavior. Triggers (ie, a SMS/MMS reminder) were also provided as part of the mScreening intervention, so that research participants did not only change in terms of their knowledge, attitudes, and/or beliefs about cervical cancer and its screening, but were also actively engaged in the utilization of the Pap test.

### Limitations

While the results of this study are very promising, there are some limitations. First, the sample size was relatively small and we used a quasi-experimental study design. Further research is needed to validate the effectiveness of the mScreening intervention with a larger sample of Korean American women using a rigorous research design, such as a randomized controlled trial. Second, our study was not designed to explore what would be the optimum time interval for program delivery. We delivered the mScreening intervention over a 7-day period. The study participants’ feedback during the post-test interviews was that a shorter intervention period would have been better. Investigations comparing the 7-day text-message program with a shorter intervention (eg, 5 days or 3 days) may determine if a briefer time may still be sufficient to bring about behavior change. However, we also postulate that an individually tailored intervention with a longer time period may be more effective for those with more barriers. Third, the ideal intervention medium is not yet known. Further research is needed to determine if it is beneficial to individually tailor the length of the intervention and if a more effective medium may result in greater behavior change (such as an interactive smartphone app). Additional investigations should examine the utility of delivering the text-message program via mobile application (mobile app), which utilizes a password function to protect participants’ confidentiality and privacy. The efforts to find the best intervention medium (text vs mobile app) and the most appropriate length of intervention (1 week vs fewer days) will create an intervention that is more effective in promoting cervical cancer screening and prevention while at the same time increasing participants’ satisfaction.

### Conclusions

Our findings revealed that mobile technology is a promising tool to increase both knowledge about cervical cancer and receipt of the Pap test. This study provides evidence for the feasibility, acceptability, and satisfaction of the mScreening intervention. Given the widespread use of mobile phones (98%) and smartphones (83%) among young adults [39], a mobile phone-based health intervention could be a cost-effective method of reaching hard-to-reach populations with tailored, individual messages that cover broad content areas and overcome restrictions to place and time of delivery. Our developed model could be expanded for delivery to different age groups of Korean American women to promote additional types of cancer screening, such as colonoscopy or mammogram. It could also be used with other underserved minority groups. Vietnamese, Hmong, and Laotian American women face similar barriers to cancer screening and report high cervical cancer incidence and mortality [40-42]. It is likely that these populations may also benefit from a similarly tailored intervention approach. Given emerging technological developments, effective interventions that could be adapted to efficiently disseminate culturally appropriate health information and promote positive health behavior changes would broadly impact the social determinants of health disparities in hard-to-reach, vulnerable populations.

## Abbreviations

FBM: Fogg’s Behavior Model
MMS: multimedia message service
mScreening: mobile phone text-message-based Screening
SMS: short message service
